# Time-Synchronized Convergence Control for n-DOF Robotic Manipulators with System Uncertainties

**DOI:** 10.3390/s24185986

**Published:** 2024-09-15

**Authors:** Duansong Wang, Gang Zhang, Tan Zhang, Jinzhong Zhang, Rui Chen

**Affiliations:** College of Electrical and Photo Electronic Engineering, West Anhui University, Lu’an 237012, China; wangduansong@hrbeu.edu.cn (D.W.); zhanggang@wxc.edu.cn (G.Z.); 42000028@wxc.edu.cn (T.Z.); zhangjinzhongz@126.com (J.Z.)

**Keywords:** time-synchronized control, finite time, rehabilitation robot, ratio persistence

## Abstract

A time-synchronized (TS) convergence control method for robotic manipulators is proposed. Adversely to finite-time control, a notion of time-synchronization convergence is introduced based on the ratio persistence property, which can ensure that all system components converge simultaneously in a finite time. Firstly, a robust disturbance observer is constructed to be compatible with the time-synchronized control framework and precisely estimate system uncertainties. Furthermore, we design a (finite) time-synchronized controller to ensure that all states of the robotic manipulator simultaneously converge to an equilibrium point, irrespective of initial conditions. Stability analysis shows the feasibility of the proposed TS control method. At last, simulations are performed with a two-link rehabilitation robotic system, and the comparison results indicate its superiority.

## 1. Introduction

With the continuous evolution of technology, robotic manipulators have emerged as crucial components in modern life. Specifically, in the medical domain, rehabilitation robots assist patients in recovering muscle functionality, enhancing exercise capabilities, and elevating their quality of life through precise control and operation [1,2,3].

Numerous contributions have been made in the control of robotic manipulators, spanning physical applications and theoretical research work [4,5,6]. In [7], a finite-time control (FTC) approach is introduced for a rehabilitation robot system. This method achieves faster convergence speeds compared to conventional sliding mode control (SMC) methods and mitigates the singular problem. In [8,9], a new sliding surface is developed for the servo system and underactuated flexible joint robot, respectively, the chattering problem is alleviated, and the system states and lumped uncertainties are estimated by the cascaded observer. Further, adaptive SMC is proposed in [10] for the motor system, which overcomes the challenges of model nonlinear and parameter perturbations. Significantly, the adaptive gain can adjust according to the system uncertainties. Additionally, in [11], authors focused on the observer design for the air conditioning system, and FTC stability was achieved. An adaptive neural network controller, in conjunction with an integral barrier Lyapunov function, is devised in [12] for the purpose of physical human–robot interactions. This controller is employed to perform tracking tasks, mitigate system uncertainties, and enhance tracking performance. An adaptive control method is designed in [13] for robot manipulators to estimate unknown parameters and relax the condition of persistent excitation with finite-time convergence. However, the convergence time of FTC depends on initial states and cannot achieve TS convergence performance.

Fixed-time control (FxTC) offers the benefit of allowing the system to set a predetermined time that is not influenced by the initial states. Additionally, users have the flexibility to predefine the time according to their specific requirements [14,15,16]. In order to deal with model uncertainties under the framework of fixed-time control, the neural network is introduced to enhance system robustness in [17]. To achieve fixed-time control while constraining tracking errors of robotic systems, a new error conversion mechanism is designed in [18]. To achieve reduced tracking errors, increased robustness, and quicker response times compared to finite-time control, ref. [19] introduces a fixed-time adaptive fuzzy backstepping control approach for robot manipulators. In [20], a robust controller is introduced with a cascaded observer to reduce the noise effect, with the method being demonstrated on a hardware platform. While numerous advancements have been documented in the domain of rehabilitation robot control within the contexts of FTC and fixed-time control, it is noteworthy that neither approach can achieve TS stability performance, particularly for systems with unknown dynamics.

To obtain TS control performance, coupling error is constructed among different joints in [21,22]. A neural network synchronous strategy is used in [23]. Drawing inspiration from FTC and the ratio persistence property, the notion of TS control was initially developed by Li in 2021 [24]. In this study, a TS control approach for the Euler–Lagrange system is formulated, integrating the norm-normalized sign function (NSF) and FTC technology to ensure simultaneous consensus among all system states, with the criteria for TS stability being elucidated. Later discussions in [25] delve into TS control and its stability analysis, leading to the conclusion that FTS in conjunction with ratio persistence is equivalent to TS stability. Subsequently, in [26], an FxTC methodology is proposed for spacecraft operations, where under the influence of the observer, TS control is achieved. In [27], a TS controller is presented for a vessel, guiding all system states to an equilibrium point, demonstrating TS control efficiency through simulation results. The research outlined above highlights the pivotal role of the NSF in the stability analysis of TS control.

Inspired by the above discussion, a TS convergence control method for robotic manipulators with system unknown dynamics is studied using the property of ratio persistence, which is different from previous work on TS control by designing a synchronization error. The primary contributions of this study are summarized as follows:

(1) A multivariable disturbance observer (MDOB) is designed based on the super-twisting structure to precisely observe the value of system uncertainties and adapt to the framework of TS stability;
(2) A TS control strategy is proposed for robotic manipulators to ensure simultaneous convergence of system states, which is different from the previous research and relaxes the construction of the TS error among joints.
(3) The singularity issue is addressed, and an analytical method for evaluating TS convergence performance is introduced, employing a contradiction strategy.

The structure of this article is formulated as follows. Some useful preliminaries and the control problem are introduced in Section 2. The control design including the observer and TS control is shown in Section 3. Simulation verification and a summary are outlined in Section 4 and Section 5, respectively. Finally, a conclusion is given in the last section.

## 2. Problem Formulation and Preliminaries

Before proceeding, some lemmas and definitions are provided to help understand the following content.

### 2.1. Preliminaries

Consider a dynamic system described as follows:

(1) $$\dot{x}=f\left(x\right),$$

where $f:\Omega \to R^{n}$ is a continuous function with an open neighborhood around the origin, $f\left(0\right)=0,x\in \Omega \subset R^{n}$.

**Definition** **1** ([24])**.** *The system (1) is TS stability if for all initial states $x_{i}\left(0\right)$, all the system states convergence simultaneously at the time T, i.e., $x_{i}\left(t\right)\neq 0$, when t<T; $x_{i}\left(t\right)=0$, when t≥T.*

**Remark** **1.** *The merits of TS can meet the requirement of high-precision control tasks such as grasping movements, multi-legged robots, and aerospace tasks [28,29].*

**Lemma** **1** ([25])**.** *For the state x described by (1), if*

(2) $$\frac{x}{∥x∥}=\xi \frac{f(x)}{∥f(x)∥},x\neq 0$$

*holds, then, x is ratio persistent, where ξ is the direction of ratio persistent, and it can be 1 or −1.*

**Lemma** **2** ([25])**.** *Consider a Lyapunov function V(x), the state x described by (1) is TS stability and the settling time is $T\left(x_{0}\right)\leq V{\left(x_{0}\right)}^{\left(1-\alpha \right)}/\left(c\left(1-\alpha \right)\right)$, if*

(1) *There is a constant c>0,0<α<1, satisfying $\dot{V}\left(x\right)\leq -cV{\left(x\right)}^{\alpha }$, i.e., finite-time stability;*
(2) *State x is ratio persistent.*

**Figure 1:**
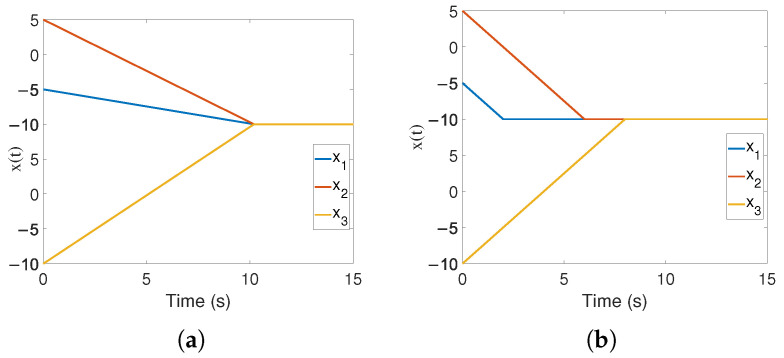
The difference between TS control and FTC. (**a**) TS control performance. (**b**) FTC performance.

**Remark** **2.** *It shows that TSstability=FTC+ratiopersistence, its basic characteristic is that all states converge simultaneously, which is the most essential difference from existing FTC/FxTC and predefined time control. Simulation examples can be seen in Figure 1a,b.*

**Remark** **3.** *From Lemma 2, it can be seen that TS control can be regarded as a special case of FTC, and the difference between them is that TS stability is stricter and requires ratio persistence.*

### 2.2. Problem Formulation

Consider a robotic manipulator system described by the following model [30]:

(3) $$M\left(q\right)\ddot{q}+C\left(q,\dot{q}\right)\dot{q}+G\left(q\right)=\tau -J{\left(q\right)}^{T}f\left(t\right)+\tau_{d},$$

where $q\in R^{n},\dot{q}$ and $\ddot{q}$ represent position, velocity, and acceleration, respectively. $M\left(q\right)\in R^{n\times n}$ is an inertia matrix, and it is a symmetry matrix that satisfies $M{\left(q\right)}^{T}=M\left(q\right)$, with $C\left(q,\dot{q}\right)\in R^{n\times n}$ and $G\left(q\right)\in R^{n}$ representing the Coriolis-centripetal and gravitational matrices, respectively. Control input is denoted by $\tau \in R^{n}$, which will be designed later, and Jacobian matrix $J\left(q\right)\in R^{n\times n}$. f(t) is a nonlinear term that represents system uncertainties; $\tau_{d}$ represents external disturbances.

This article’s control goal is to develop a TS control law τ for a robotic manipulator system described by (3), such that the system states *q* track the desired trajectory $q_{d}={\left[q_{d1},q_{d2},\cdots ,q_{dn}\right]}^{T}$; all state components not only arrive to an equilibrium point in a finite time but also in a same moment, that is to say, the (finite) time-synchronized stability. Meanwhile, an observer compatible with the time-synchronized stability should be designed to strengthen the system robustness.

## 3. Control Design

**Figure 2:**
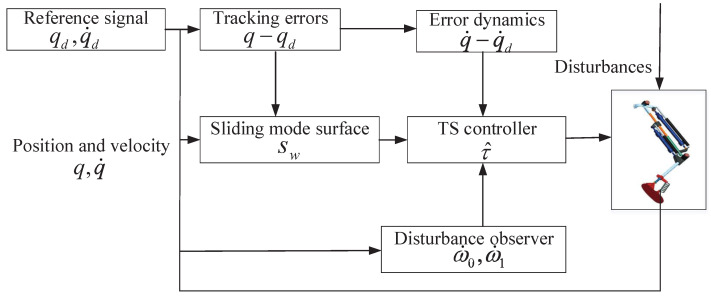
The TS control scheme.

An MDOB is first developed to compensate for the lumped uncertainties, which is also compatible with the framework TS control. The TS controller is introduced based on the Lyapunov stability. Lastly, performance analysis is given, which theoretically proves the characteristic of TS convergence. Figure 2 displays the control block diagram.

### 3.1. Disturbance Observer Design

Let $x_{1}=q$, $x_{2}=\dot{q}$; thus, system (3) can be transformed to the following format:

(4) $$\begin{array}{cc} {\dot{x}}_{1}= & x_{2},\\ {\dot{x}}_{2}= & -M{\left(q\right)}^{-1}C\left(q,\dot{q}\right)x_{2}-M{\left(q\right)}^{-1}G+\tau_{w}\\ \; & +M{\left(q\right)}^{-1}\tau , \end{array}$$

where $\tau_{w}=M{\left(q\right)}^{-1}\left(-J{\left(q\right)}^{T}f\left(t\right)+\tau_{d}\right)$.

**Assumption** **1.** *For a known constant ρ, the system uncertainties satisfies $∥{\dot{\tau }}_{w}∥\leq \rho$.*

**Remark** **4.** *For Assumption 1, it is reasonable to assume external disturbances are bounded in practice [31,32], and ensure that Formula (7) is meaningful.*

By converting (4) into the state space form, one has

(5) $$\dot{X}=f\left(X\right)X+Hu+\Delta +G_{m},$$

where

$$X={\left[x_{1}^{\prime },x_{2}^{\prime }\right]}^{T},f\left(X\right)=\left[\begin{array}{cc} 0_{n\times n} & I_{n\times n}\\ 0_{n\times n} & -M^{-1}C \end{array}\right],H=\left[\begin{array}{c} 0_{n\times n}\\ M^{-1} \end{array}\right]$$

$$\Delta =\left[\begin{array}{c} 0_{n\times 1}\\ M^{-1}\left(\tau_{d}-J^{T}f\right) \end{array}\right],G_{m}=\left[\begin{array}{c} 0_{n\times 1}\\ -M^{-1}G \end{array}\right],u=\left[\begin{array}{c} 0_{n\times 1}\\ \tau \end{array}\right].$$

Motivated by [33,34], the following super-twisting observer is designed to estimate system uncertainties.

(6) $$\begin{array}{cc} {\dot{\omega }}_{0}= & -l_{1}\frac{\omega_{0}-X}{∥\omega_{0}{-X∥}^{\frac{1}{2}}}-l_{2}\left(\omega_{0}-X\right)+f\left(X\right)X+Hu+G_{m}+\omega_{1},\\ {\dot{\omega }}_{1}= & -l_{3}\frac{\omega_{0}-X}{∥\omega_{0}-X∥}-l_{4}\left(\omega_{0}-X\right), \end{array}$$

where $l_{1},l_{2},l_{3},l_{4}$ are the constant parameters and will be designed later, $\omega_{0}$ is the approximation of the state, and $\omega_{1}$ is the estimation value of uncertainties.

Define ${\tilde{\omega }}_{0}=\omega_{0}-X$ and ${\tilde{\omega }}_{1}=\omega_{1}-\Delta$; thus, the observation error can be obtained, as follows:

(7) $$\begin{array}{cc} {\dot{\tilde{\omega }}}_{0}= & -l_{1}\frac{{\tilde{\omega }}_{0}}{∥{\tilde{\omega }}_{0}{∥}^{\frac{1}{2}}}-l_{2}{\tilde{\omega }}_{0}+\omega_{1},\\ {\dot{\tilde{\omega }}}_{1}= & -l_{3}\frac{{\tilde{\omega }}_{0}}{∥{\tilde{\omega }}_{0}∥}-l_{4}{\tilde{\omega }}_{0}-\dot{\Delta }. \end{array}$$

**Lemma** **3.** *If Assumption 1 holds, the observer errors ${\tilde{\omega }}_{0}$ and ${\tilde{\omega }}_{1}$ can be driven to zero in finite time $T_{ob}$ by choosing the proper values of $l_{1},l_{2},l_{3},l_{4}$ under the effect of the system (7); moreover, $T_{ob}\leq \frac{2}{c}V_{0}^{\frac{1}{2}}$ and $V_{0}=X_{0}^{T}PX_{0}$, where*

(8) $$X_{0}={\left(\frac{{\tilde{\omega }}_{0}^{T}\left(0\right)}{∥{\tilde{\omega }}_{0}{\left(0\right)}^{\frac{1}{2}}∥},{\tilde{\omega }}_{0}^{T}\left(0\right),{\tilde{\omega }}_{1}^{T}\left(0\right)\right)}^{T},$$

(9) $$c=\frac{2\lambda_{\max}\left(P\right)}{\lambda_{\min}\left(\Omega \right)\sqrt{\lambda_{\max}\left(P\right)}},$$

*and $P\in R^{3*3}$ and $\Omega \in R^{3*3}$ are positive definite matrices, respectively.*

To avoid redundancy, the proof is omitted here. For details, please refer to Proposition 1 in [33].

### 3.2. Time-Synchronized Control Design

The desired position is defined as $q_{d}=\left[q_{d1},q_{d2},\cdots ,q_{dn}\right]\in R^{n}$; thus, one can obtain the tracking error

(10) $$z_{1}=q-q_{d},$$

For convenience, let $x_{1}=z_{1}$, $x_{2}={\dot{z}}_{1}$; thus, one has

(11) $$\begin{array}{cc} {\dot{x}}_{1}= & x_{2},\\ {\dot{x}}_{2}= & M{\left(q\right)}^{-1}\left(\tau +\gamma -J{\left(q\right)}^{T}f\left(t\right)+\tau_{d}\right)-{\ddot{q}}_{d}. \end{array}$$

where $\gamma =-C\left(q,\dot{q}\right)\dot{q}-G\left(q\right)$.

Next, a sliding mode variable concerned with the normalized function is constructed as

(12) $$s=x_{2}+{\left\lfloor x_{1}\right\rceil }^{\alpha },$$

where $\frac{1}{2}<\alpha <1$ is an adjustable control parameter. $\left\lfloor x_{1}\right\rceil$ is a normalized sign function and is defined as follows:

(13) $$\left\lfloor x_{1}\right\rceil =\left\{\begin{array}{c} \frac{x}{∥x∥},x\neq 0,\\ \;\;0,\;\;x=0, \end{array}\right.$$

where ∥·∥ represents $L_{2}$ the norm of ·.

Its exponential form is described as

(14) $${\left\lfloor x_{1}\right\rceil }^{\beta }={∥x∥}^{\beta }\left\lfloor x_{1}\right\rceil ,$$

where β is a positive constant.

**Remark** **5.** *The NSF ⌊x⌉ is used to construct a sliding mode surface; it is essential to the stability analysis of finite-time-synchronized control.*

The TS control law is designed as

(15) $$\begin{array}{cc} \tau = & -M({\left\lfloor s\right\rceil }^{\alpha }+\left(\alpha -1\right)∥x_{1}{∥}^{\alpha -3}x_{1}x_{1}^{T}x_{2}\\ \; & +∥x_{1}{∥}^{\alpha -1}x_{2}-{\ddot{q}}_{d})-\gamma \\ \; & +J{\left(q\right)}^{T}f\left(t\right)-\tau_{d}. \end{array}$$

Considering the lumped unknown uncertainties term $-J{\left(q\right)}^{T}f\left(t\right)+\tau_{d}$, it can be precisely observed by the system of (6). Then, TS control is given by

(16) $$\begin{array}{cc} \hat{\tau }= & -M({\left\lfloor s\right\rceil }^{\alpha }+\left(\alpha -1\right)∥x_{1}{∥}^{\alpha -3}x_{1}x_{1}^{T}x_{2}\\ \; & +∥x_{1}{∥}^{\alpha -1}x_{2}-{\ddot{q}}_{d})-\gamma -M*\omega_{1}. \end{array}$$

**Theorem** **1.** *Considering the manipulator system characterized by (3), under the effect of TS control (16) and the multivariable super-twisting observer (6), the system is TS stability. All the state components of $z_{11},z_{12},\ldots ,z_{1n}\in z_{1}$ can converge at the same time.*

**Proof.**  The differentiation of *s* along time is

(17) $$\begin{array}{cc} \dot{s} & =M^{-1}\tau -M^{-1}\left(-\gamma +J{\left(q\right)}^{T}f\left(t\right)\right)\\ \; & -{\ddot{q}}_{d}+∥x_{1}{∥}^{\alpha -1}x_{2}+\left(\alpha -1\right){∥x_{1}∥}^{\alpha -3}x_{1}x_{1}^{T}x_{2}\\ \; & =-{\left\lfloor s\right\rceil }^{\alpha }-\omega_{1}+\tau_{w}. \end{array}$$

Since the system (6) can precisely observe the value $\tau_{w}$ at set time $T_{ob}$, $\omega_{1}=\tau_{w}$; thus, we have

(18) $$\dot{s}=-{\left\lfloor s\right\rceil }^{\alpha },t\geq T_{ob}.$$

A candidate Lyapunov function is chosen as

(19) $$V_{1}=s^{T}s.$$

Take the differential of Lyapunov function $V_{1}$ along time and substitute (16) into it; thus, one can obtain

(20) $${\dot{V}}_{1}=-2s^{T}{\left\lfloor s\right\rceil }^{\alpha }=-2V_{1}^{\frac{1+\alpha }{2}}.$$

The settling time of FTC is $T_{1}=\frac{V_{1}{\left(s_{0}\right)}^{\frac{1-\alpha }{2}}}{1-\alpha }$, where $s_{0}$ is the initial value of *s*.

Then, by using Lemma 2 and (18), one can get

(21) $$\frac{\dot{s}}{∥\dot{s}∥}=\frac{-{\left\lfloor s\right\rceil }^{\alpha }}{{∥-\left\lfloor s\right\rceil }^{\alpha }∥}=-\frac{{s∥s∥}^{\alpha -1}}{{∥s∥s∥}^{\alpha -1}∥}=-\frac{s}{∥s∥},$$

Therefore, *s* holds the property of ratio persistence; the conclusion of *s* holds the finite TS stability, which can be drawn from Lemma 2. When *s* converges to zero, according to (12), one knows that

(22) $$x_{2}=-{\left\lfloor x_{1}\right\rceil }^{\alpha }.$$

Next, to prove the stability of $x_{1}$, a Lyapunov function is chosen, as follows:

(23) $$V_{2}=x_{1}^{T}x_{1}.$$

The differential of $V_{2}$ can be written as

(24) $${\dot{V}}_{2}=-2x_{1}^{T}{\left\lfloor x_{2}\right\rceil }^{\alpha }=-2V_{2}^{\frac{1+\alpha }{2}}.$$

Hence, the conclusion of system (11) is finite-time stability and the settling time is $T_{2}=\frac{V_{2}{\left(x_{2}\left(0\right)\right)}^{\frac{1-\alpha }{2}}}{1-\alpha }$, which can be drawn from Lemma 2.

To obtain the synchronized convergence performance of $x_{1}$, the ratio persistence property of $x_{1}$ is needed. From (22), one has

(25) $$\frac{{\dot{x}}_{1}}{∥{\dot{x}}_{1}∥}=\frac{x_{2}}{∥x_{2}∥}=\frac{-{\left\lfloor x_{1}\right\rceil }^{\alpha }}{∥-{\left\lfloor x_{1}\right\rceil }^{\alpha }∥}=\frac{-x_{1}{∥x_{1}∥}^{\alpha -1}}{∥x_{1}∥x_{1}{∥}^{\alpha -1}∥}=-\frac{x_{1}}{∥x_{1}∥},$$

which can demonstrate that $x_{1}$ holds the property of ratio persistence from Lemma 1, leading to TS convergence of the state $x_{1}$. This completes the proof. □

### 3.3. Performance Analysis

To further clarity the time-synchronized convergence performance, a contradictory method is used.

Suppose that any two state elements $x_{1i},x_{1j}\in x_{1}\in R^{n}$ and $x_{1i},x_{1j}\neq 0$ arrive at an equilibrium at a different time, $t_{i}$ and $t_{j}$; thus, $t_{i}<t_{j}$, i.e., $x_{1i}{{|}_{t=t_{i}}=0,x_{1j}|}_{t=t_{j}}=0$, respectively. According to (25), this yields

(26) $${\dot{x}}_{1}=-\frac{∥{\dot{x}}_{1}∥}{∥x_{1}∥}x_{1}=Cx_{1},$$

where *C* is a constant and $C=-\frac{∥{\dot{x}}_{1}∥}{∥x_{1}∥}$.

Then, by calculating the derivation of $x_{1i}/x_{1j}$, one has

(27) $$\frac{d}{dt}\frac{x_{1i}}{x_{1j}}=\frac{{\dot{x}}_{1i}x_{1j}-x_{1i}{\dot{x}}_{1j}}{x_{1j}^{2}},$$

Then, by substituting (26) into (27), one can obtain

(28) $$\frac{d}{dt}\frac{x_{1i}}{x_{1j}}=0.$$

Therefore, $x_{1i}=d_{ij}x_{1j}$, where $d_{ij}$ is a constant.

From (24), $x_{1}$ is finite-time stability; moreover, for $t_{i}\neq t_{j}$,

(29) $$\begin{array}{c} \lim_{t\to t_{i}^{-}}x_{1i}=0,\lim_{t\to t_{j}^{-}}x_{1j}=0. \end{array}$$

Combined with (28), $\lim_{t\to t_{i}^{-}}x_{1j}\left(t\right)=0$ is directly obtained and contradicts the hypothesis. Therefore, any two state elements $x_{1i},x_{1j}\in x_{1}\in R^{n}$ and $x_{1i},x_{1j}\neq 0$ arrive at an equilibrium at the same time instant. This completes the analysis of time-synchronized convergence.

**Remark** **6.** *It should be noticed that a singularity problem exists due to the presence of powers −3 and −1 in (15). To avoid a singularity problem, one should follow the nonsingular TS control law.*

(30) $$\begin{array}{cc} \hat{\tau }= & -M\left({\left\lfloor s\right\rceil }^{\alpha }+{\dot{s}}_{w}-{\ddot{q}}_{d}\right)+C\left(q,\dot{q}\right)\dot{q}+G\left(q\right)\\ \; & -M*\omega_{1}, \end{array}$$

*with the sliding mode $s_{non}=x_{2}+s_{w}$, and*

(31) $$s_{w}=\left\{\begin{array}{c} {\left\lfloor x_{1}\right\rceil }^{\alpha },\mathrm{if}\;s^{*}=0\;\mathrm{or}\;s^{*}\neq 0,∥x_{1}∥>\xi ,\\ \mu_{1}x_{1}+\mu_{2}{\left\lfloor x_{1}\right\rceil }^{4},\mathrm{if}\;s^{*}\neq 0,∥x_{1}∥\leq \xi , \end{array}\right.$$

*$s^{*}=x_{2}+{\left\lfloor x_{1}\right\rceil }^{\alpha }$, $\mu_{1}=\frac{4-\alpha }{3}{∥\xi ∥}^{\alpha -1}$,$\mu_{2}=\frac{\alpha -1}{3}{∥\xi ∥}^{\alpha -4}$, ξ represents a small constant.*

**Theorem** **2.** *Considering the robotic manipulator system described by (3), under the effect of the TS method (30) and the multivariable super-twisting observer (6), the system is TS stability, and the singularity problem can be avoided. Meanwhile, all the state components of $z_{11},z_{12},\cdots ,z_{1n}\in z_{1}$ can converge at the same time.*

**Proof.**  The process is similar to the proof of Theorem 1 by choosing $V=s_{non}^{T}s_{non}$. The TS convergence performance analysis is the same as in the Section 3.2. □

## 4. Simulation Results

Simulations are conducted on a robot system to demonstrate the TS control’s effectiveness. In addition, a comparison was made with finite-time control.

### 4.1. Validation of the TS Control

**Figure 3:**
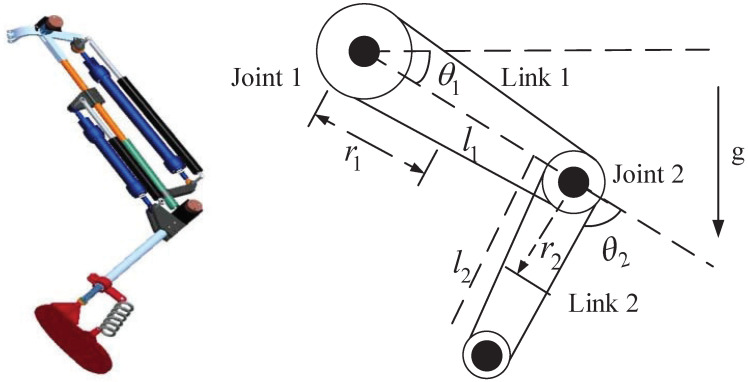
Model of the knee rehabilitation robotic manipulator.

In this section, numerical simulations are conducted on a two-link knee rehabilitation robotic system, illustrated in Figure 3. $I_{i}$, $m_{i}$, and $l_{i}$ represent the moment of inertia, mass, and length of link *i*, respectively. $l_{ci}$ denotes the distance between the joint i−1 to *i* with i=1,2. For the details of the knee rehabilitation robotic system’s parameters, please refer to [30]. Here, we define $q={\left[q_{1},q_{2}\right]}^{T}$. The control parameter α for TS is set to 0.6. The control program runs on a computer (AMD Ryzen 5 5600H, Radeon Graphics 3.30 GHz) using Matlab 2024b.

(32) $$q=\left[\begin{array}{c} q_{1}\\ q_{2} \end{array}\right]=\left[\begin{array}{c} \theta_{1}\\ \theta_{2} \end{array}\right]$$

**Figure 4:**
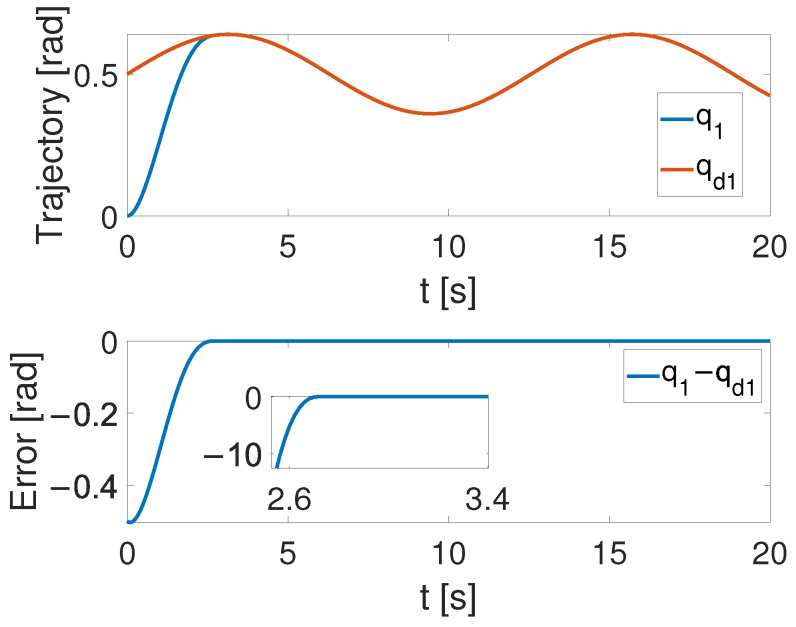
The tracking performance of joint 1 under the effect of TS control.

**Figure 5:**
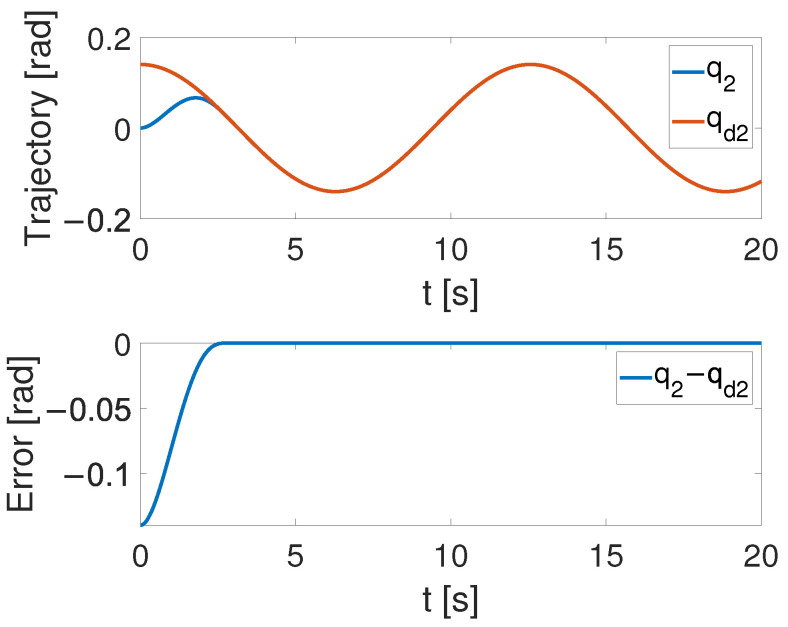
The tracking performance of joint 2 under the effect of TS control.

**Figure 6:**
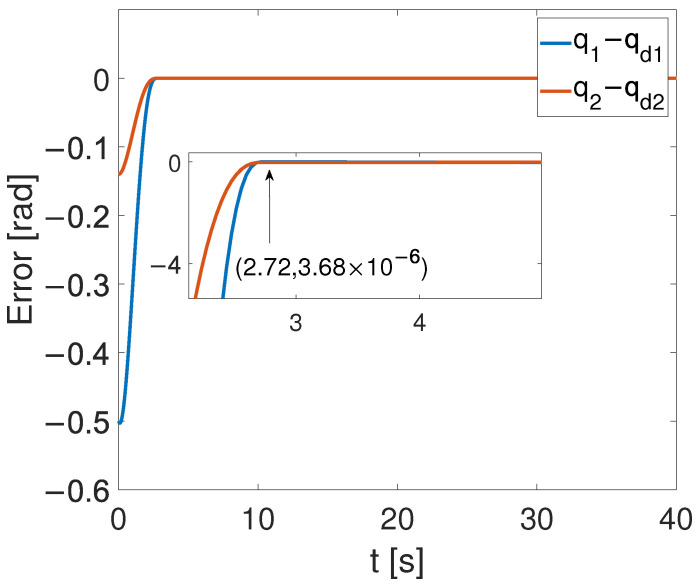
Error convergence curves of the two joints.

**Figure 7:**
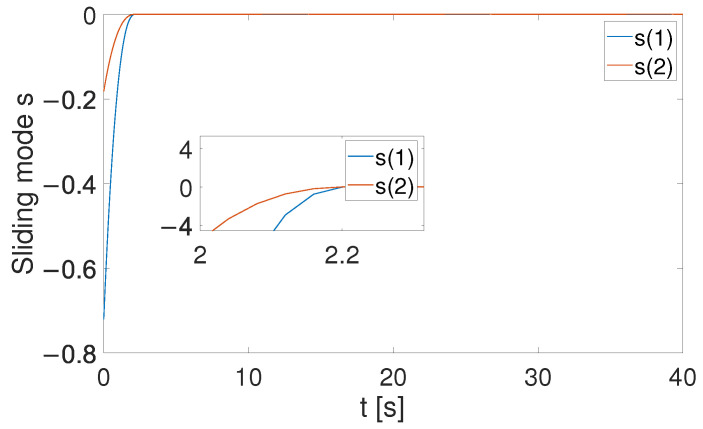
Sliding surface in (31).

**Figure 8:**
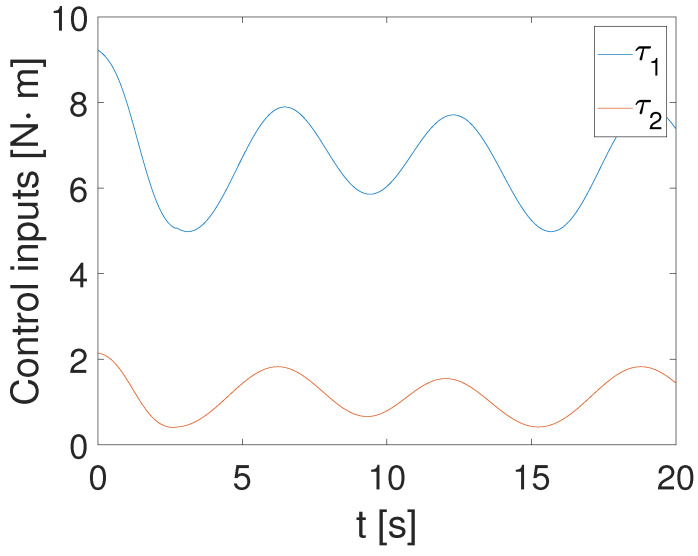
Control input of the TS method.

**Figure 9:**
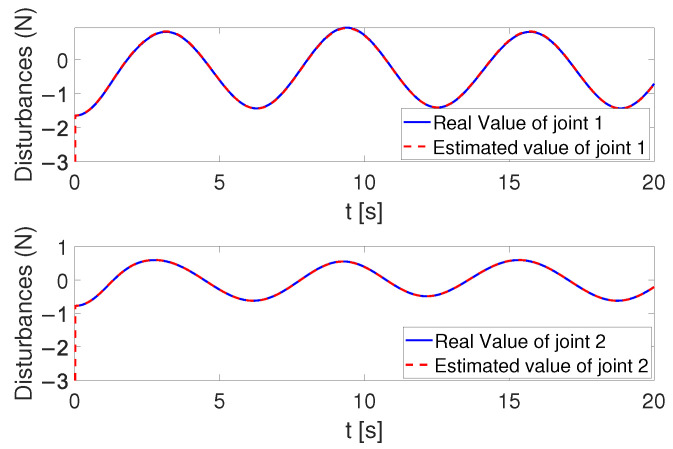
The real and estimated value of the observer.

Figure 4, Figure 5 and Figure 6 show that the suggested TS control is valid. Figure 4 and Figure 5 illustrate the trajectory tracking performance of the two joints. In Figure 4, it is observed that the joint follows the desired trajectory well after several seconds. Even with a large initial error value, the error converges within a few seconds under the influence of the time-synchronized control. So does the joint 2 depicted in Figure 5. Figure 6 shows that the errors of the two joints converge simultaneously at 2.72 s, even though the initial errors are different. Figure 7 displays the changing of the sliding surface, revealing that s(1) of joint 1 and s(2) of joint 2 converge simultaneously at the time constant t=2.2, which reflects that the sliding surface first converges, followed by the tracking error. This also demonstrates the unique characteristic of the time-synchronized convergence performance. Figure 8 is the control signal. It is obvious that there is no traditional chattering problem. Figure 9 shows the estimated and real value of the proposed observer, reflecting that after the first several seconds, the estimated value curve coincides with the actual value curve.

### 4.2. Comparison with the Existing Work

To confirm that the proposed time-synchronized control is effective, the control performance is evaluated in comparison to the other state-of-the-art controller proposed in [35], which is based on a neural network and barrier function, and its form is as follows:

(33) $$\begin{array}{cc} \tau = & M({\dot{\alpha }}_{1}-k_{2}x_{2}-k_{B2}^{-1}k_{B1}x_{1}-0.5k_{B2}x_{2}-{\hat{W}}^{T}\phi \left(x\right)), \end{array}$$

where $\alpha_{1}=-k_{1}x_{1}+{\dot{q}}_{d}$. Control parameters are set as $k_{1}=100,\;k_{2}=100,\;k_{B1}=0.1,\;k_{B2}=1$.

**Figure 10:**
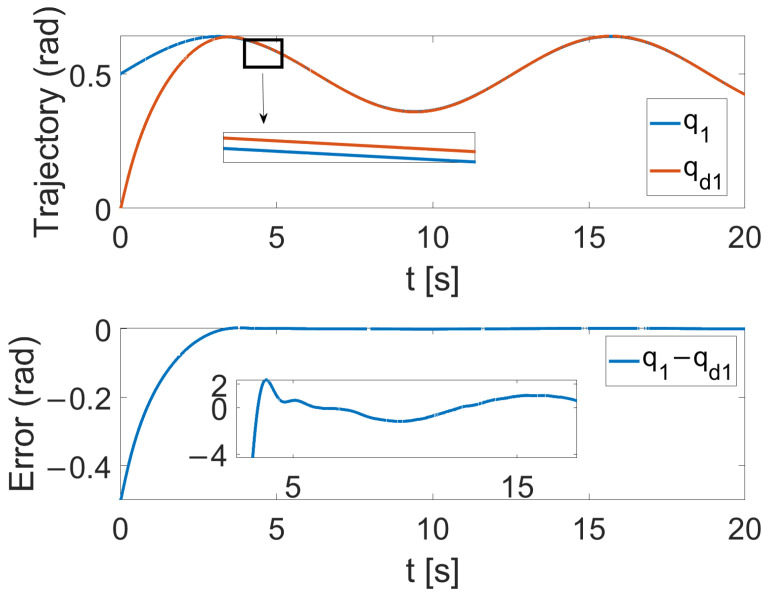
Tracking performance of joint 1 with FTC.

**Figure 11:**
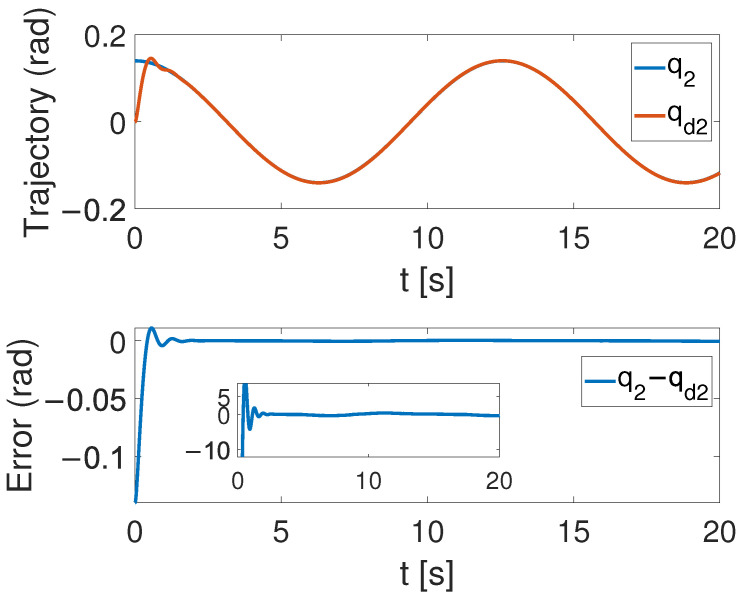
Tracking performance of joint 2 with FTC.

**Figure 12:**
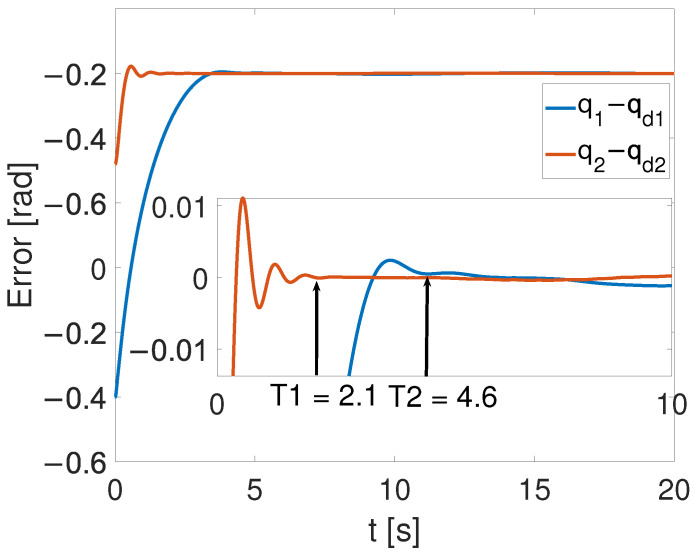
Error convergence curve with FTC.

Figure 10, Figure 11 and Figure 12 show the results of the simulation. Figure 10 illustrates that, despite the achievement of the tracking task, a static error remains. In Figure 11, one can see that the overshoot phenomenon exists in the tracking curve. Furthermore, in Figure 12, the convergence times for the two joints are, respectively, $T_{1}=2.1$ s and $T_{2}=4.6$ s. By comparing Figure 6 and Figure 12, the time-synchronized convergence performance is directly proved.

**Remark** **7.** *The control parameters involved in the proposed TS control are $\alpha ,\mu_{1},\mu_{2},\xi$, and $l_{1}-l_{4}$. For comparative finite-time control, the parameters included are $k_{1},k_{2},k_{B1}$, and $k_{B2}$. The function of these values can be seen as proportional gain. The larger the value, the faster the system response; however, this is not necessarily better. When it exceeds a certain range, it will cause the system to diverge and become unstable, making it unable to complete corresponding control tasks. Here, we determine these values using the trial and error method.*

## 5. Discussion

It is shown in Figure 5 that by using the proposed TS control, the trajectory errors of the two joints converge to 3.68 $\times \;10^{-6}$ at the time constant T=2.72 s simultaneously, while the tracking errors of the two joints converge to the equilibrium point at $T_{1}=2.1$ s and $T_{2}\;=\;4.6$ s, respectively, with the finite-time controller. This demonstrates the advantages of the TS controller. These super merits can meet high-precision control tasks, such as the ones in aerospace and multi-agent systems, especially for multi-legged robots and grasping movements.

Though TS convergence performance was achieved, it is worth noticing that under the effect of FTC, joint 1 converged to the equilibrium point at T=2.1 s, which is faster than T=2.72 s. In other words, though the proposed TS method can drive all joints to converge simultaneously at the same moment, the limitation is that the faster joint may have to wait for the slower one. This drawback can be solved by combining predefined time control with the TS control proposed here.

## 6. Conclusions

Being different from previous TS control studies by designing a synchronization error of robotic manipulators, this work explored nonsingular TS control with system uncertainties directly based on FTC and ratio persistence, which simplified the procedure of control design, allowing us to obtain super performance. Additionally, a multivariable super-twisting observer was devised, capable of accurately estimating system uncertainties within a settling time. Time-synchronized convergence performance was assessed through the contradiction method. The proposed TS control method was compared with other strategies, verifying the TS method’s effectiveness. Our future work will explore the extension of the TS convergence method to other MIMO systems, including multi-agent systems, unmanned surface vehicles (USVs), and more. 

## Data Availability

The data presented in this study are available upon request from the corresponding author.
